# Age dependence of brain oxygen metabolism in adults assessed by 3D constrained quantitative BOLD MRI

**DOI:** 10.1016/j.neuroimage.2026.121967

**Published:** 2026-04-28

**Authors:** Kathryn M. Jaroszynski, Sina Dindarian, Hyunyeol Lee, John A. Detre, Felix W. Wehrli

**Affiliations:** a Department of Radiology, Perelman School of Medicine, University of Pennsylvania, Philadelphia, PA, USA; b Department of Bioengineering, School of Engineering and Applied Sciences, University of Pennsylvania, Philadelphia, PA, USA; c School of Electronic and Electrical Engineering, Kyungpook National University, Republic of Korea; d Department of Neurology, Perelman School of Medicine, University of Pennsylvania, Philadelphia, PA, USA

**Keywords:** Brain oxygen metabolism, Brain mapping, Aging, Blood flow, Oxygenation, 3D MRI, Quantitative BOLD

## Abstract

Global and regional variations in cerebral oxygen metabolism are known to be associated with both healthy aging and the early stages of neurodegeneration. To quantify age effects, a recently developed 3D constrained qBOLD protocol was implemented to determine voxelwise OEF, integrated with ASL-derived CBF measurements to compute CMRO_2_. Thirty-four healthy adults (23–87 years) were examined to identify global and regional patterns of cerebral oxygen metabolism across younger (<35, n = 15) and older (>50, n = 19) adults. Whole-brain OEF was greater in older than in younger adults (40.2 ± 5.1 % vs 34.5 ± 4.6 %, P = 0.002), whereas CBF was lower (39.5 ± 10.3 vs 48.3 ± 8.3 mL/100*g*/min, P = 0.011), resulting in comparable CMRO_2_ (144.0 ± 31.8 vs 150.7 ± 30.0 μmol/100*g*/min, P = 0.537). Across participants, OEF increased by 1.5 % and CBF declined by 2.1 % per decade. Regionally, OEF was greater in older adults within medial temporal lobe (MTL) (37.4 ± 5.9 % vs 32.2 ± 5.1 %), hippocampus (37.2 ± 7.0 % vs 29.1 ± 6.1 %), amygdala (38.3 ± 8.6 % vs 27.9 ± 6.1 %), thalamus (40.5 ± 6.4 % vs 32.5 ± 7.1 %), striatum (42.7 ± 5.3 % vs 36.2 ± 5.5 %), frontal (38.7 ± 5.2 % vs 34.7 ± 5.0 %), parietal (43.9 ± 5.1 % vs 38.2 ± 5.5 %), and occipital (40.7 ± 6.3 % vs 35.1 ± 5.3 %) lobes, all P < 0.05. CMRO_2_ differed between older vs younger subjects only in the amygdala (126.6 ± 35.9 vs 97.5 ± 31.6 μmol/min/100 g, P = 0.01). Exploring regional heterogeneity, MTL and its substructures exhibited reduced OEF relative to whole-brain averages in both groups. Overall findings indicate that cerebral oxygen metabolism remains largely preserved throughout adulthood. The combined qBOLD–ASL technique offers a robust framework for detecting regional variations in brain oxygen metabolism, characterizing both normative cerebrovascular aging and possibly early stages of neurodegeneration.

## 1. Introduction

Oxygenation and perfusion are essential physiological processes that support neural function and metabolic homeostasis in the human brain (Catchlove et al., 2018). While comprising only 2 % of the adult body weight, the brain accounts for roughly 20 % of the body’s total energy budget (Cunnane et al., 2020). Global and regional differences in cerebral oxygen utilization and blood flow have been associated with both healthy aging and the early stages of neurodegenerative diseases (Catchlove et al., 2018; Aanerud et al., 2012; De Vis et al., 2015; Ishii et al., 1996; Jiang et al., 2020; Jiang et al., 2023; Pantano et al., 1984; Taghvaei et al., 2025; Tohgi et al., 1998; Yang et al., 2023). Furthermore, studies indicate that metabolic demands vary across brain structures reflecting the heterogeneity of cerebral oxygen extraction and blood flow (Ishii et al., 1996; Jiang et al., 2023; Henriksen et al., 2021; Ito et al., 2023). Therefore, the ability to accurately assess brain oxygenation-related parameters offers a window into investigating physiological patterns in health and disease.

Positron emission tomography (PET) using ^15^O-labeled radiotracers remains the reference standard for quantifying these parameters (Fan et al., 2020). However, PET’s limited accessibility, complex set-up, dependence on short-lived isotopes and arterial blood sampling have restricted its use in large-scale or routine assessments (Fan et al., 2020). As a result, magnetic resonance imaging (MRI)-based methods have gained increasing attention for their ability to non-invasively estimate cerebral oxygenation and perfusion metrics using endogenous contrast mechanisms (Yang et al., 2023; An and Lin, 2000; Cho et al., 2018; Davis et al., 1998; Englund et al., 2020; He and Yablonskiy, 2007; Jain et al., 2010; Lee and Wehrli, 2022; Lu and Ge, 2008).

Several MRI oximetry methods have emerged in recent years to assess global CMRO_2_ and OEF. The T_2_-relaxation-under-spin-tagging (TRUST) technique utilizes venous spin-labeling to obtain the T_2_ relaxation time of the blood, which is then converted to venous oxygen saturation (Y_v_) via a calibration plot (Lu and Ge, 2008). MOTIVE (Metabolism of Oxygen via T_2_ Interleaved Velocity Encoding) is another T_2_-based technique which simultaneously measures blood velocity and blood water T_2_ (Deshpande et al., 2022). An alternative approach is susceptometry, which exploits the magnetic susceptibility differences between venous blood and the surrounding tissue (Wehrli, 2024). This method, termed ‘OxFlow’, quantifies global organ oxygen saturation and blood flow (Jain et al., 2010). However, while these methods are useful to obtain whole-brain metabolic data, they do not provide any regional information.

Three MRI techniques evolved during the past two decades have shown potential in quantifying oxygen extraction fraction in the human brain regionally, relying on the blood oxygenation level dependent (BOLD) phenomenon (Ogawa et al., 1990). The first is referred to as calibrated BOLD (cBOLD) (Davis et al., 1998) requiring calibration of the BOLD MRI signal through gas breathing challenges (hypercapnia or hyperoxia). However, the cBOLD method only provides fractional changes in O2 metabolism in response to a hypercapnic or hyperoxic stimulus, rather than absolute values, unless both stimuli are used, which is referred to as dual calibrated BOLD (Gauthier et al., 2012). This limits the method’s clinical utility due to a complicated experimental protocol and patient discomfort. The second is referred to as ‘quantitative BOLD’ (qBOLD) (An and Lin, 2000; He and Yablonskiy, 2007; Yablonskiy et al., 2013), which does not require signal calibration. The third is quantitative susceptibility mapping (QSM), which provides a calibration-free quantification of the oxygenation dependent parenchymal magnetic susceptibility (Zhang et al., 2017; Zhang et al., 2015). The recognition that neither qBOLD nor QSM provide adequate information for oxygen extraction fraction (OEF) quantification in the presence of non-heme originated magnetic perturbers (e.g. brain iron), led more recently, to methods that combine QSM and qBOLD techniques into a hybrid model (QQ), as the two approaches provide complementary information that minimizes error for oxygen parameter estimation and allow for rapid 3D imaging (Cho et al., 2018) without the need to acquire data at two different metabolic states (Zhang et al., 2015). While QQ methods have shown good agreement with PET-derived OEF values and have demonstrated sensitivity to physiological changes across cortical and subcortical structures (Cho et al., 2021; Elanghovan et al., 2025), they do not directly measure R_2_’, the reversible portion of the transverse relaxation rate that, under certain conditions, is linearly proportional to deoxygenated blood volume (DBV) and Y_v_ (He and Yablonskiy, 2007). They also rely on clustering or machine learning techniques to reduce noise within the model (Cho et al., 2021; Cho et al., 2022; Cho et al., 2020). Some of the present authors developed a method termed “constrained” qBOLD MRI, which, in addition to incorporating QSM, directly estimates R_2_’, and CBV_v_ as prior information, thereby minimizing noise and improving parameter quantification (Lee and Wehrli, 2022). The method, dubbed ‘constrained qBOLD’ has since been validated against calibrated BOLD and whole-brain oximetry techniques, as well as in response to several physiologic stimuli (Jaroszynski et al., 2025; Lee et al., 2024).

Together these advances underscore the feasibility of characterizing regional oxygenation profiles in healthy individuals using constrained qBOLD MRI. As studies increasingly emphasize region-specific metabolic phenotypes (Yang et al., 2023; Henriksen et al., 2021; Ito et al., 2023; Lee and Wehrli, 2022; Jaroszynski et al., 2025), there is a growing need to establish normative baselines of oxygenation and perfusion across anatomically distinct areas in the human brain. Understanding these patterns across a range of ages may facilitate the identification of early degenerative changes.

The aim of this study was to characterize global and region-specific patterns of cerebral oxygenation and perfusion across healthy adults covering a range of ages by means of constrained qBOLD MRI. Evaluation of regional variability can help determine whether MRI-based assessments reveal significant physiological differences across brain structures in the absence of clinical disease, thereby contributing to the growing effort to establish robust, noninvasive biomarkers of both normative and at-risk brain physiology.

## 2. Materials and methods

### 2.1. Parameter estimation

#### 2.1.1. Constrained qBOLD

The constrained qBOLD technique, henceforth referred to simply as ‘qBOLD’, consists of two sequences for OEF estimation; ‘Alternating Unbalanced Steady-state-free-precession FID and Echo’ (AUSFIDE) and ‘Velocity-Selective Venous Spin Labeling’ (VS-VSL) (Lee and Wehrli, 2022). AUSFIDE consists of alternating SSFP-FID and ECHO multi-echo signal components, which over multiple repetition times (TRs) decay with rate constants $R_{2}+R_{2}’=({R_{2}}^{⋆})$ and $R_{2}-R_{2}’$, respectively (Lee and Wehrli, 2021). In this manner, R_2_ and R_2_’ can be estimated from magnitude processing, and relative magnetic susceptibility (Δχ) and macroscopic field inhomogeneity (ΔB_0_) are obtained through processing of the phase data (Lee and Wehrli, 2021). The second sequence, VS-VSL, employs slab-selective saturation and spatially nonselective inversion pulses to suppress signal from arterial blood and cerebrospinal fluid (Lee and Wehrli, 2020). During a velocity-selective pulse train, control/tag images are subtracted from one another to suppress static tissue and isolate the venous blood signal (Lee and Wehrli, 2020). In this way, a map of the venous cerebral blood volume (CBV_v_) can be obtained directly from the control/tag difference.

The parameters estimated from both AUSFIDE and VS-VSL (R_2_, R_2_’, Δχ, ΔB_0_, and CBV_v_) are then input as prior constraints into the qBOLD optimization problem (from Lee and Wehrli, 2022):

(1) $$argmin(\Theta )\sum_{TE}{|y(TE)-\Xi (\Theta ,TE)|}^{2}+w{|\Delta \chi -\Psi (\Theta )|}^{2}+p{|R_{2}^{\prime }-Y(\Theta )|}^{2}$$

Here, y represents the AUSFIDE signals (SSFP-FID and SSFP-ECHO) at each echo time (TE), and the corresponding model is represented by Ξ. The symbol Θ describes the set of unknown parameters, which include venous oxygen saturation (Y_v_), deoxygenated blood volume (DBV), R_2_’nh (non-heme), and non-blood tissue susceptibility (χ_nb_) (Lee and Wehrli, 2022). The model Ψ comprises Δχ, and accounts for all four pools of susceptibility within a voxel (deoxygenated arterial and venous blood, fully oxygenated blood, and tissue) (Lee and Wehrli, 2022). Finally, *Y* decomposes R_2_’ into heme and non-heme iron contributions, and w and *p* are regularization parameters. This nonlinear inverse optimization problem is solved on a voxel-by-voxel basis to obtain parametric maps of the unknown parameters. Full explanation of this model can be found in Lee (Lee and Wehrli, 2022).

#### 2.1.2. CBF and CMRO_2_ quantification

When combined with a measurement of cerebral blood flow (CBF), the qBOLD data yield quantitative maps of the cerebral metabolic rate of oxygen consumption (CMRO_2_). Here, a pseudo-continuous arterial spin labeling (pCASL) sequence (Vidorreta et al., 2017) was used to map CBF in units of ml/min/100 g of brain tissue. Brain density was assumed to be ~1.05 *g*/ml. Combining these data with an estimation of OEF (in percent) from qBOLD, CMRO_2_ is calculated in units of μmol/min/100 g of brain tissue using Fick’s Principle (Xu et al., 2009):

(2) $$CMRO_{2}=CBF\cdot C_{a}\cdot S_{a}O_{2}\cdot OEF$$

Here, C_a_ is the oxygen carrying capacity of arterial blood, calculated by multiplying hematocrit by the oxygen binding capacity of red blood cells (C_rbc_ = 19.93 μmolO_2_/ml). S_a_O_2_ was assumed to be 98 % and hematocrit was determined from a finger prick test (Hemocue© 201+, Sweden).

### 2.2. Study design and data acquisition

#### 2.2.1. Participants

A cross-sectional cohort of 34 healthy adults (age range: 23–87 years, 15 subjects < 35, 19 subjects > 50) was included in this study. All participants were free of neurological or psychiatric disorders and provided written informed consent. Privacy rights of all subjects were observed. The study protocol was performed in compliance with relevant laws and institutional guidelines and approved by the University of Pennsylvania’s Institutional Review Board (Protocol #857,342). Subjects over the age of 50 were given the Mini Mental State Examination (MMSE) to confirm normal cognition (Folstein et al., 1975). Subjects were asked to abstain from caffeine the day of the study. Hematocrit (Hct) was measured from a finger-prick blood sample on the day of MRI acquisition (Hemocue©, Sweden). A subset of these subjects’ data was used in a prior publication (Jaroszynski et al., 2025) (N = 7). A summary of subject demographics is shown in Table 1.

#### 2.2.2. MRI data acquisition

**Fig. 1.**
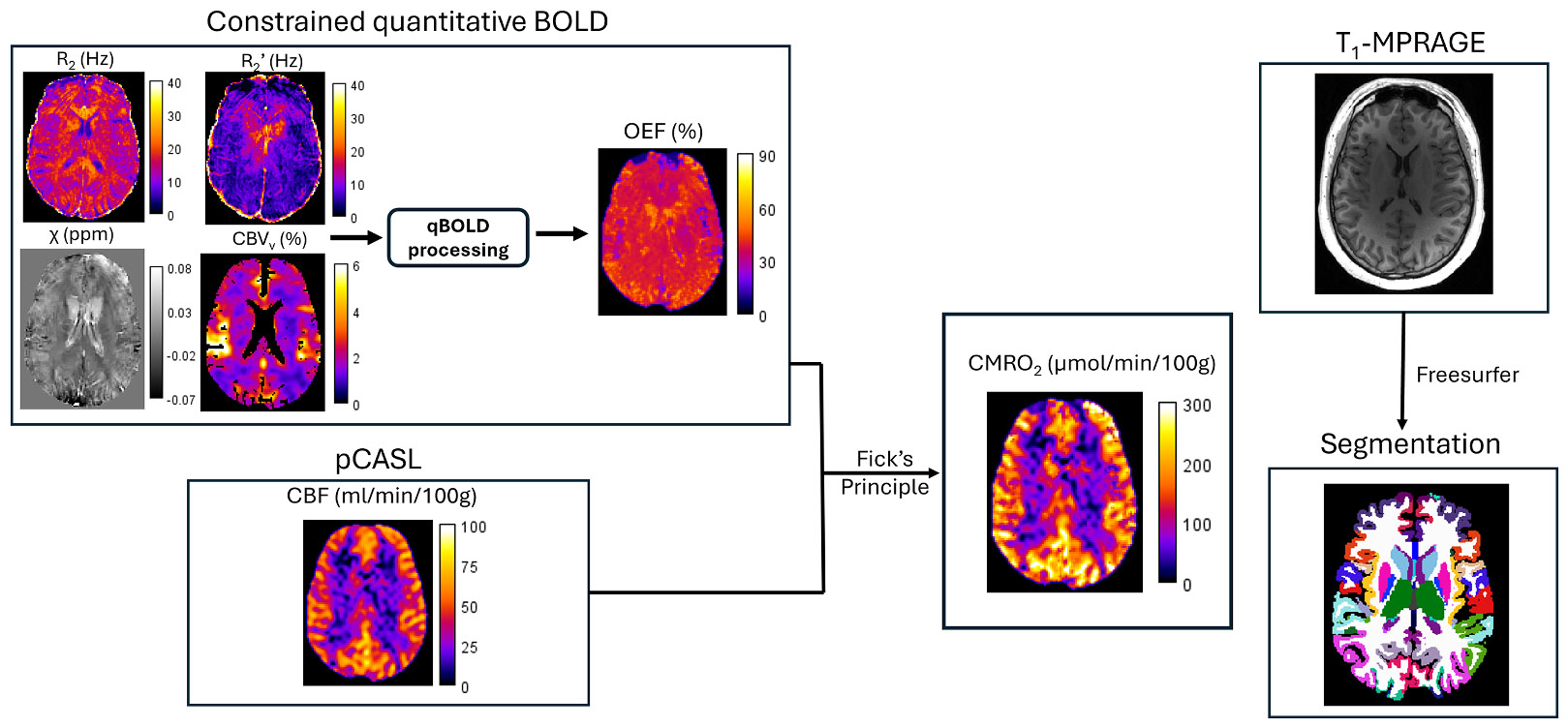
Overview of the constrained qBOLD protocol. Prior information from AUSFIDE and VS-VSL sequences are input into the qBOLD optimization problem to quantify oxygen extraction fraction (OEF). When combined with a cerebral blood flow (CBF) measurement from a pCASL sequence, CMRO_2_ can be calculated using Fick’s Principle. The T1-MPRAGE collected was input into the FreeSurfer algorithm for automatic segmentation of cortical and subcortical regions. Images shown are from a 25-year-old male subject.

All imaging was performed on a Siemens MAGNETOM Prisma 3T whole-body scanner (Siemens, Germany) using a 32 or 64-channel head coil. The total protocol, including qBOLD, pCASL, and structural imaging was approximately 20 min long. For OEF quantification, the AUSFIDE (Lee and Wehrli, 2021) parameters were as follows: FOV = 240 × 240 × 120 mm^3^, matrix size = 160 × 160 × 40, first TE (FID) = 1.6 ms, first TE (ECHO) = 2.2 ms, repetition time (TR) = 30 ms, number of echoes = 17, echo spacing = 1.5 ms. The total scan time for this sequence is 8 min. For VS-VSL (Lee and Wehrli, 2020), the parameters were: FOV = 240 × 240 × 180 mm^3^, matrix size = 72 × 72 × 60, TR = 3 s, saturation time (TS) = 1.65 s, and inversion time (TI) = 1.14 s, resulting in a scan time of 3.3 min. The 3D pseudo-continuous ASL sequence (Vidorreta et al., 2017) had an FOV of 240 × 240 × 140 mm^3^, matrix size equal to 68 × 68 × 40, post-labeling delay of 2 s, and 6 control/tag pairs, totaling a scan time of 4.3 min. High-resolution anatomic images were obtained with a sagittal 3D T1-weighted magnetization-prepared rapid gradient-echo (MPRAGE) sequence with an isotropic voxel resolution of approximately 1.0 × 1.0 × 1.0 mm^3^ and a scan time of 4.5 min for sub-region segmentation and brain volume determination. An overview of imaging and analysis protocol is given in Fig. 1.

### 2.3. Data analysis

All data reconstruction and post-processing for AUSFIDE and VS-VSL was done in MATLAB (Mathworks©, MA).

#### 2.3.1. Segmentation of anatomic regions of interest (ROIs)

Gray and white matter automatic segmentations were done using the statistical parametric mapping (SPM12) software in MATLAB (Friston, 2011). To reduce partial volume effects from gray matter spilling, the white matter segmentation was constrained by morphological erosion using a 1 voxel structural element to remove boundary voxels. Automatic segmentation of cortical and subcortical structures was performed using FreeSurfer (version 7.4.1) (Fischl, 2012) with the Desikan–Killiany–Tourville (DKT) (Klein and Tourville, 2012) or Destrieux (Destrieux et al., 2010) atlas applied to each participant’s T1-weighted image. In this manner, anatomic masks for global gray and white matter, as well as cortical and subcortical structures of interest were obtained. All segmentation masks were registered to the first echo image of the FID from AUSFIDE and resampled to match the resolution of the qBOLD data (1.5 × 1.5 × 3 mm). Prior to regional averaging, all parametric maps were masked to retain only voxels within the specified anatomic ROI and those yielding physiologically plausible OEF, CBF or CMRO_2_ values, defined as positive and finite values. Voxel values below the 5th percentile or above the 95th percentile were excluded to minimize contamination by noise or fitting artifacts. This percentile filtering was applied within each ROI rather than at the whole-brain level to ensure local data integrity. ROI extraction and volumetric calculations were implemented in Python (version 3.12.7) using NiBabel (version 5.3.2), NumPy (version 1.26.4), and Pandas (version 2.2.2).

#### 2.3.2. Statistical analysis

All statistical analyses were performed using Python (version 3.12.7) and SPSS (version 22). The significance threshold was set at *α* = 0.05 (two-tailed). Data normality was assessed using the Shapiro–Wilk test, with parametric tests applied when normality was met and equivalent nonparametric tests otherwise. To control for multiple comparisons across tests, the Benjamini–Hochberg false discovery rate procedure was applied at *q* = 0.05. Adjusted *q*-values are reported, and changes in significance after correction are described. Figures and data visualizations were generated in Python using the Matplotlib and Seaborn libraries.

##### Group comparisons.

For each predefined region of interest (ROI), mean values of oxygen extraction fraction (OEF, %), cerebral blood flow (CBF, mL/100 g/min), and cerebral metabolic rate of oxygen (CMRO_2_, μmol O_2_/100 g/min) were calculated after within-ROI 5th–95th percentile trimming to minimize the influence of outliers. Group differences between younger (21–35 years) and older (50–90 years) adults were tested using independent-samples *t*-tests when data were normally distributed or Mann–Whitney U tests otherwise.

Within-subject regional differences from whole-brain averages were assessed separately within each age group using paired-samples t tests or Wilcoxon signed-rank tests as appropriate. 95 % confidence intervals (CIs) were obtained from test outputs.

##### Volumetric analyses.

Regional and global brain volumes were compared between younger and older adults using independent-sample t tests or Mann–Whitney U tests, according to nature of distribution. To assess regional variation in brain structure, gray-to-whole-brain and white-to-whole-brain volume ratios were computed, providing relative measures of tissue composition across age groups.

##### Correlation and regression analyses.

Associations between physiological variables and age as well as structural variables were examined using Pearson’s correlation and linear regression analyses. Specifically, global and regional OEF, CBF, and CMRO_2_ were correlated with age across all participants to assess metabolic changes associated with aging.

To account for potential sex and head size-related physiological differences, regression models for metabolic parameters versus age included sex and normalized brain volume as covariates. Intracranial volume (ICV) was calculated for each participant as the sum of gray matter, white matter, and cerebrospinal fluid volumes derived from structural MRI segmentation. To account for variability in brain volume due to constitutional differences in head size and sex, regional and global brain volumes were normalized to ICV and expressed as percentages of ICV. The multiple linear regression model was then fitted of the form:

(3) $$Metric=\beta_{0}+\beta_{1}\cdot Age+\beta_{2}\cdot Sex+\beta_{3}\cdot NormalizedVolume$$

For each region, a multiple linear regression model was fitted with age as the independent variable and each physiological parameter as the dependent variable. Unstandardized regression coefficients (B) and standard errors were reported, representing the absolute rate of change per year, along with 95 % confidence intervals and two-tailed p-values. Standardized coefficients (β) were also obtained to describe the strength of association independent of measurement units. Regression assumptions (linearity, homoscedasticity, and normality of residuals) were verified for all models. Simple linear regression models not including covariates were fitted for visualization, and Pearson’s correlation coefficients (*r*) and corresponding two-tailed *p*-values are reported for each region. Given the primarily descriptive nature of the study and to maintain consistency across analyses, Pearson’s correlation was applied uniformly, even where minor deviations from normality were observed. For volumetric analyses, linear regression models were fitted using ICV-normalized regional volumes as dependent variables without inclusion of additional covariates.

##### Laterality analyses.

Laterality was examined separately for metabolic and volumetric measures. For metabolic parameters, laterality indices (LI) were calculated within each age group as (Right − Left) / (Right + Left) for the hippocampus and amygdala, two key structures of the medial temporal lobe where right/left volumetric differences have been reported (Pedraza et al., 2004), but no such study has been done regarding metabolic differences between hemispheres. Mean LI values were tested against zero using one-sample *t*-tests or Wilcoxon signed-rank tests when non-normal. Effect sizes for within-subject comparisons were expressed as Cohen’s dz, calculated as the mean difference divided by the standard deviation of the paired differences. For volumetric data, the same procedure was applied, with the thalamus additionally included given its central integrative role within cortico-limbic pathways and its well-established sensitivity to age-related structural changes (Hughes et al., 2012). Positive LI values indicated rightward asymmetry, whereas negative values indicated leftward dominance. Although LI values were statistically assessed within each age group, violin plots were generated across the full cohort to illustrate the overall distribution and direction of hemispheric asymmetry. Mean LI and corresponding two-tailed *p*-values are reported.

## 3. Results

### 3.1. Sample characteristics

A total of 34 healthy adults participated in the study, comprising 15 younger (21–35 years; 44.1 %) and 19 older (50–87 years; 55.9 %) individuals. All participants successfully completed the MRI protocol and were included in the final analysis. The younger group consisted of 11 males (73.3 %) and 4 females (26.7 %), while the older group included 14 males (73.7 %) and 5 females (26.3 %), resulting in a comparable sex distribution between age groups despite the overall male predominance (χ^2^ = 0.001, P = 0.982). All participants were screened to exclude neurological, psychiatric, or systemic illness and demonstrated hematocrit levels within normal physiological ranges.

### 3.2. Effect of age on brain oxygen metabolic parameters

**Fig. 2.**
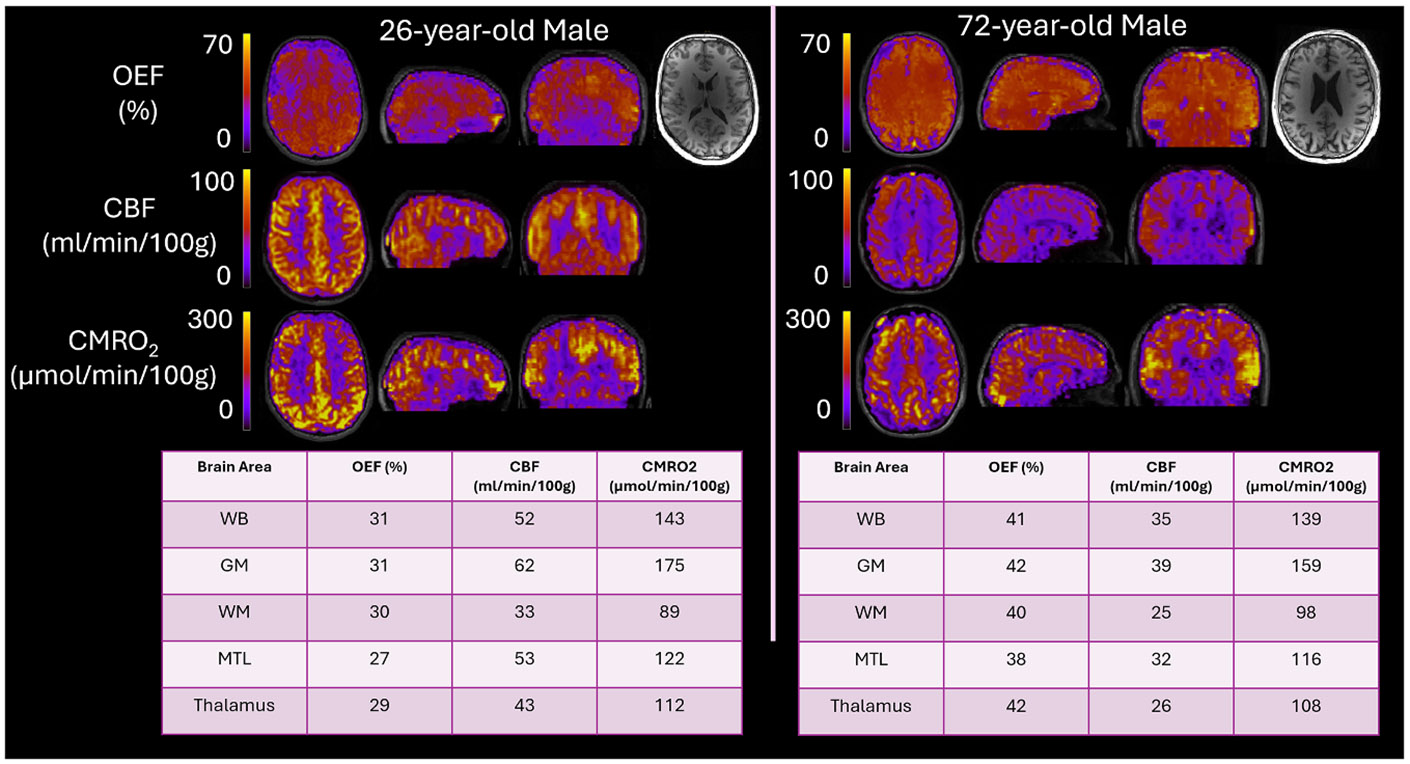
OEF, CBF, and CMRO_2_ images from two representative study subjects differing in age. Note the visually apparent greater OEF and lower CBF in the older individual, borne out by the measurements in the tables. Atrophy can also be seen in the older subject’s anatomical images shown on the right. CMRO_2_ is comparable between subjects.

Fig. 2 shows representative images from one younger (Age 26) and older (Age 72) subject. Visually, CBF is significantly lower in the older subject and OEF is noticeably higher. Whole brain values of CBF were 35 versus 52 ml/min/100 g in the older and younger subjects, respectively. Similarly, OEF values were 41 versus 31 %. However, CMRO_2_ values were similar for both ages (139 versus 143 μmol/min/100 g).

**Fig. 3.**
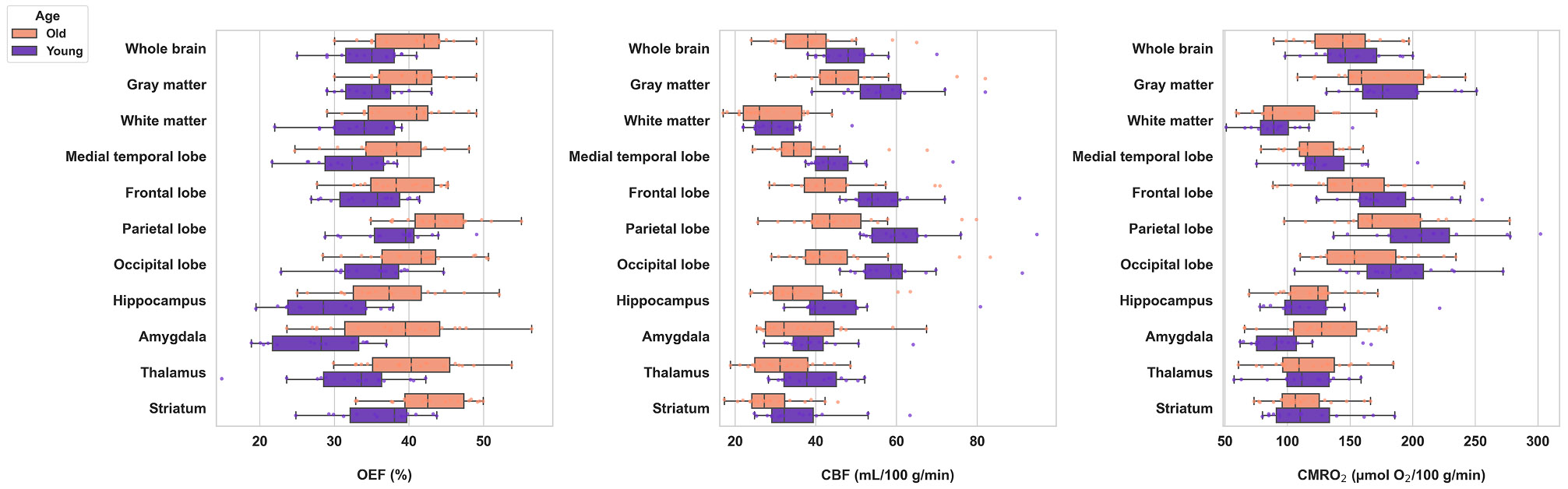
Group differences in whole-brain and regional neurometabolic parameters. Boxplots show oxygen extraction fraction (OEF), cerebral blood flow (CBF), and cerebral metabolic rate of oxygen (CMRO_2_) across whole brain and eight anatomically defined regions for younger (21–35 years) and older (50–90 years) adults. Each box represents the first (25 %) to third (75 %) quartile of the data, with the central line indicating the median. Whiskers extend to the minimum and maximum values excluding outliers, and individual data points are overlaid for each subject. Note consistently greater OEF and lower CBF in the older relative to the younger subgroup.

Mean global and regional metabolic parameters are given in Table 2. After Benjamini–Hochberg correction, all OEF differences between age groups remained significant (q < 0.03). For CBF, results were also unchanged, with all significant regions remaining significant (q < 0.04) while white matter and amygdala remained non-significant (q = 0.425 and 0.222, respectively). For CMRO_2_, the amygdala demonstrated a nominal difference between age groups (P = 0.011) that did not survive correction (q = 0.121). Across all regions, OEF was higher in older adults, and CBF was lower (Fig. 3). The distributions for CMRO_2_ were found to substantially overlap across groups in most regions, representing lesser age effects.

**Fig. 4.**
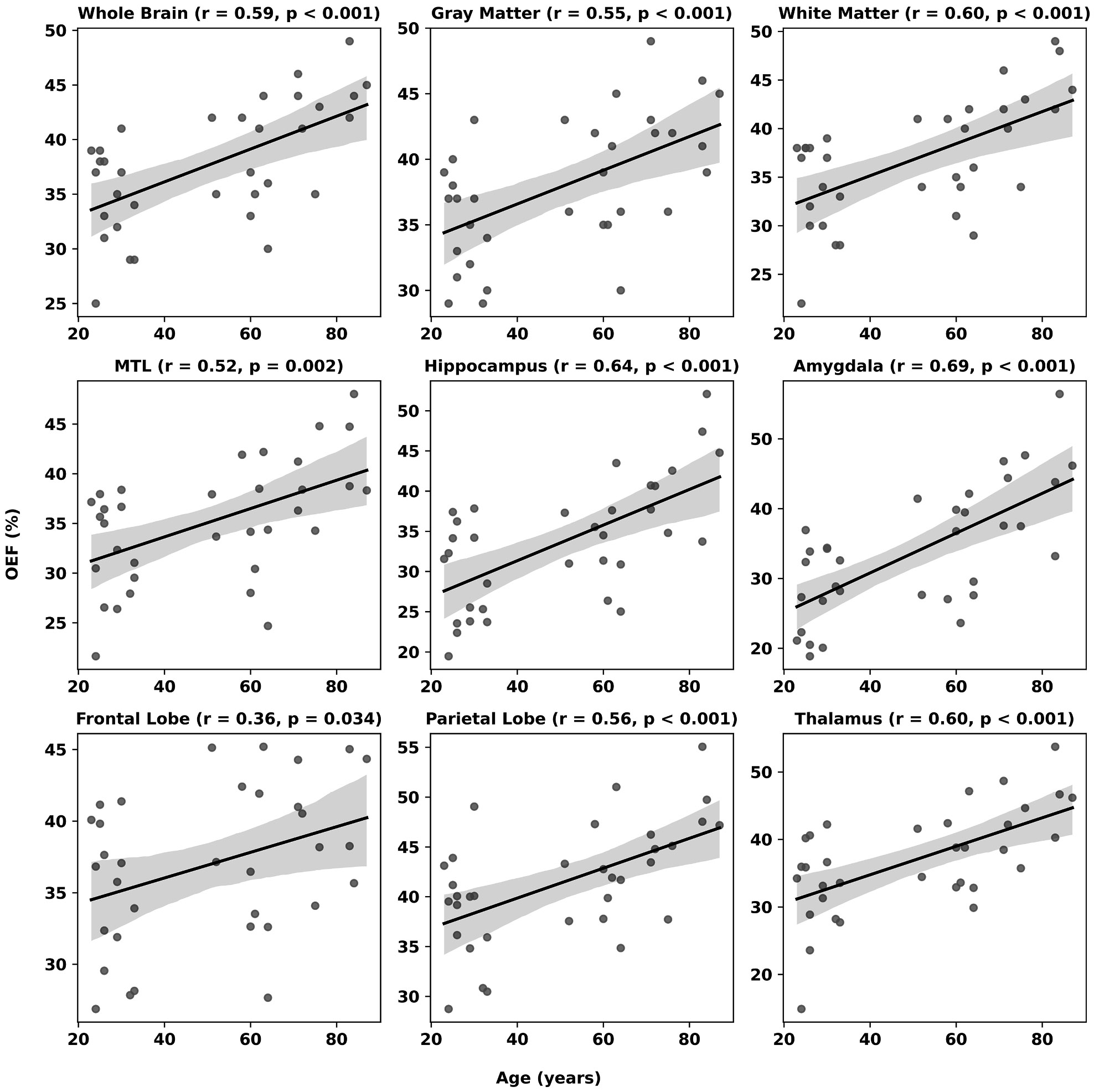
Relationship between age (years) and oxygen extraction fraction (OEF, %). Note significant positive associations for the whole brain and across all brain regions.

**Fig. 5.**
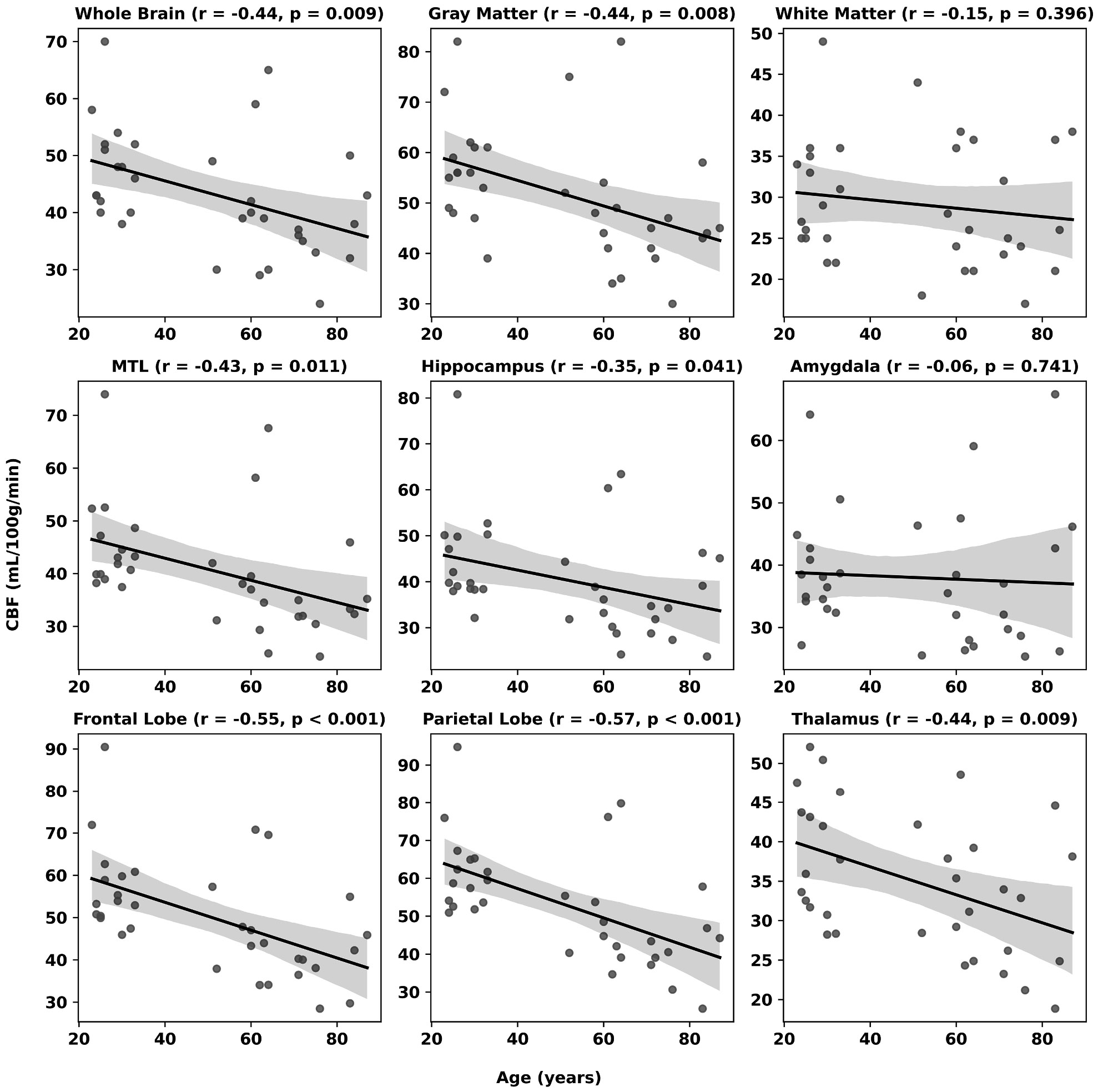
Relationship between age (years) and cerebral blood flow (CBF, mL/100 *g*/min). Note significant negative associations in the whole brain and across most brain regions.

**Fig. 6.**
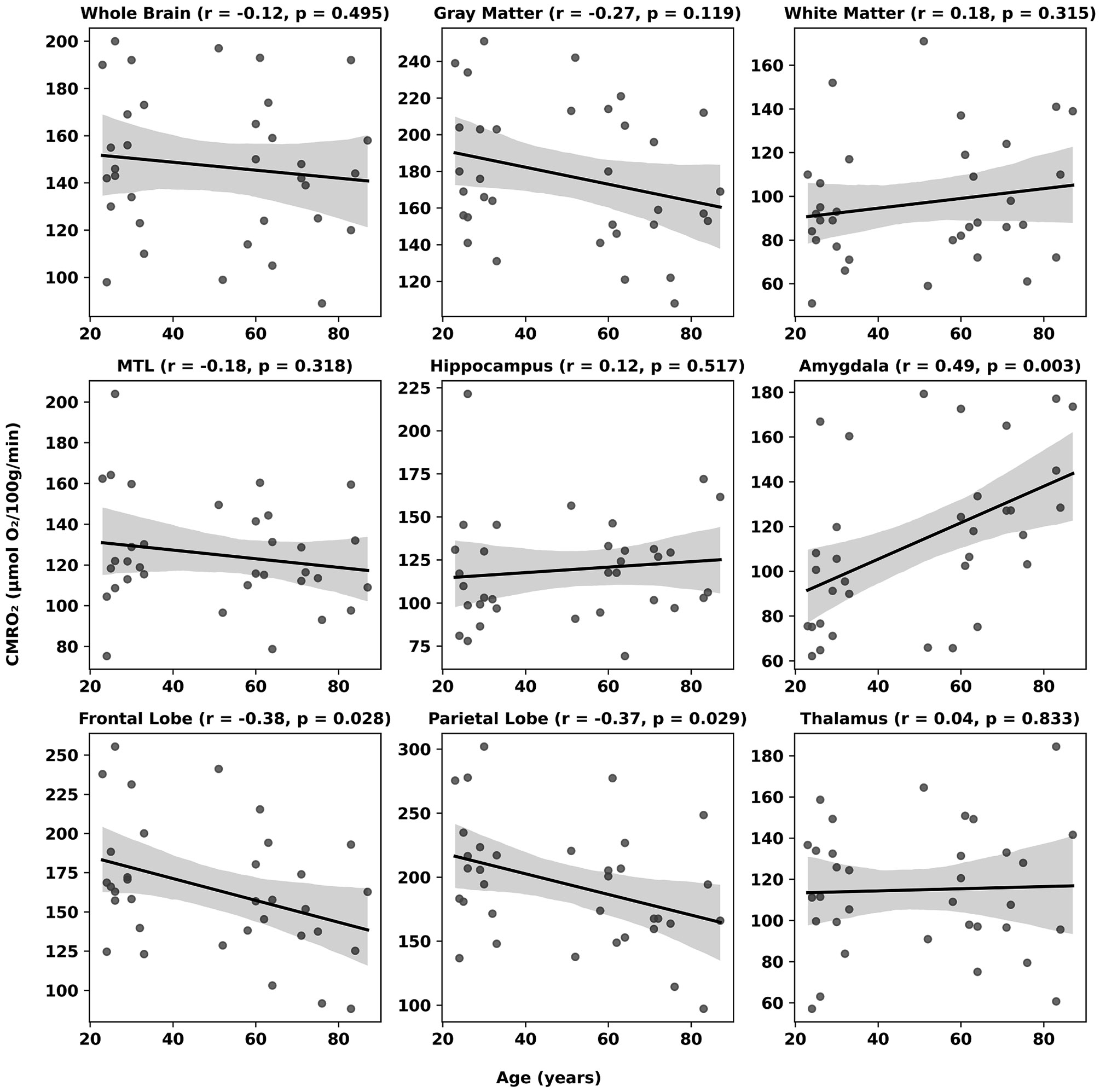
Relationship between age (years) and the cerebral metabolic rate of oxygen (CMRO_2_, μmol O_2_/100 g/min). Note significant associations in three regions, with a positive correlation in the amygdala and negative correlations in the frontal and parietal lobes, but not in remaining regions.

Across participants, OEF showed a robust age-related increase throughout cortical and subcortical regions (Fig. 4). Whole-brain OEF was found to strongly increase with age at a rate of 1.5 % per decade (r = 0.59, P < 0.001), exhibiting comparable behavior in both gray and white matter. Regionally, the largest rate of change with respect to age was observed in the amygdala, hippocampus, and thalamus, increasing at rates of 2.9 %, 2.2 %, and 2.1 % per decade, respectively (all P < 0.001). Age was inversely associated with CBF across most cortical and subcortical regions (Fig. 5), with significant declines observed globally and regionally, particularly in the frontal and parietal lobes (r = −0.55 and −0.57, P < 0.001), as well as the thalamus and medial temporal lobe. Hippocampal CBF decreased modestly with age (r = −0.36, P = 0.039), while amygdala blood flow remained relatively invariant across ages. Across regions, metabolic rates remained largely constant, with only small declines noted in frontal and parietal cortices (r ≈ −0.37 and −0.38, P < 0.05) and a modest positive correlation in the amygdala (r ≈ 0.49, P = 0.003) (Fig. 6).

Regression statistics for OEF, CBF, and CMRO_2_ across all brain regions are summarized in Table 3. Age was significantly associated with OEF across all examined regions (all P < 0.04). For CBF, age was significantly inversely associated with perfusion across most regions, except for white matter (P = 0.389) and the amygdala (P = 0.764). For CMRO_2_, nominal age-related associations were observed only in the amygdala (β = 0.83, P = 0.002), as well as in the frontal (β = −0.68, P = 0.029) and parietal (β = −0.79, P = 0.033) lobes. However, after Benjamini–Hochberg correction for multiple comparisons, only the amygdala remained significant (q = 0.016), whereas the frontal and parietal lobes associations did not survive correction (both q = 0.123). All OEF associations remained significant after correction (q < 0.04), while the age-related association in CBF for the hippocampus was no longer significant (q = 0.058). Normalized brain volume was not a significant predictor of OEF (all P > 0.08), CBF (all P > 0.12), or CMRO_2_ (all P > 0.14) in any region, indicating that inter-individual differences in brain volume do not independently explain variability in these physiological parameters beyond the effect of age. Sex, likewise, showed no significant effects in the model, except for CMRO_2_ in the amygdala (β = 30.0, P = 0.017), where higher values were observed in males. However, the age-related association in this region remained significant after adjustment (P = 0.002).

### 3.3. Regional oxygen metabolism relative to whole brain averages

Table 4 provides mean differences in OEF, CBF, and CMRO_2_ (region – whole brain) across brain regions in both age groups. Positive differences indicate that a region has higher metabolic or oxygenation values than the whole-brain average, whereas negative values indicate lower activity, reflecting normal variation in metabolic demand across brain regions. After Benjamini–Hochberg correction, all differences remained significant except for thalamic OEF in the younger group (P = 0.038, q = 0.054), and frontal OEF (P = 0.036, q = 0.072) in the older group. In the younger group, the amygdala and hippocampus showed the largest differences from whole brain OEF (−6.6 and −5.4 %). However, these differences were much less pronounced in the older subjects (−1.9 and −3.0 %). A similar trend was seen for CBF, where differences between regional (white matter, amygdala, frontal lobe, parietal lobe, occipital lobe) and global values were much smaller for the older cohort. The difference in OEF in the parietal lobe was the same in both groups (+3.7 %), and together with the striatum (+1.7 % in younger and +2.5 % in older participants), it was among the few regions that exhibited higher OEF than the whole brain. The frontal, parietal, and occipital lobes had higher CMRO_2_ in both age groups compared to whole-brain values, while all other regions exhibited lower CMRO_2_.

### 3.4. Associations between brain volume and age

Mean volumetric parameters in brain regions separated by age group are listed in Table 5. Global and regional brain volumes were generally lower in older compared to younger adults. Total brain and gray matter volumes showed significant reductions with age, while white matter volume trended lower. The proportion of gray matter relative to whole brain volume was also found to decrease with age, accompanied by a relatively higher white-matter fraction in older adults. Hippocampal volume was significantly lower in the older group, while amygdala volume only trended lower (P = 0.104). Benjamini–Hochberg correction did not alter these observations.

Linear regression (Supplementary Table 1), performed using ICV-normalized volumes, revealed a small but significant age-related decline in global brain volume (−0.02 %ICV per decade, P < 0.001). In contrast, gray (P = 0.090) and white (P = 0.108) matter volumes did not show significant associations with age. Among subcortical and neocortical regions, only the hippocampus demonstrated a significant age-related decline (−0.02 %ICV per decade, P = 0.009), whereas amygdala (P = 0.105) and thalamic (P = 0.377) volumes were not significantly associated with age.

### 3.5. Hemispheric laterality in neurometabolic and volumetric parameters

LIs for OEF, CBF, and CMRO_2_ were computed within the hippocampus and amygdala for each age group (Table 6). Across both younger and older adults, hemispheric asymmetries in metabolic parameters were minimal. Neither group showed significant laterality for CBF or CMRO_2_ in either region. The only group-specific effect was a modest leftward asymmetry in amygdalar OEF observed in older adults (P = 0.006, Z=−2.74).

When examined across all subjects, metabolic parameters remained largely symmetric, though a small but significant leftward asymmetry was detected in hippocampal OEF (P = 0.020, dz=0.42), while all other measures showed no lateral bias (Supplementary Figure 1).

Volumetric analyses revealed a similar but slightly more regionally differentiated pattern of asymmetry. Across both age groups, LIs were generally small, indicating limited hemispheric asymmetry in regional volumes. In younger adults, the amygdala and thalamus showed modest rightward and leftward asymmetries, respectively, whereas hippocampal volumes were symmetric. In older adults, significant leftward asymmetry of the thalamus and rightward asymmetry of the amygdala were observed. When comparing hemispheric volumes between groups, both hippocampal (right, P = 0.003; left, P = 0.005) and thalamic (right, P = 0.004; left, P = 0.007) volumes were significantly larger in younger adults, whereas amygdala volumes (right, P = 0.147; left, P = 0.083) did not differ significantly (Supplementary Table 2).

At the whole-subjects level, volumetric parameters exhibited a small but significant rightward asymmetry in the amygdala and a modest leftward asymmetry in the thalamus, while hippocampal volumes remained balanced across hemispheres (Supplementary Figure 2).

Finally, individual-level analyses provided further confirmation of these trends. Across participants, paired right–left comparisons demonstrated minimal hemispheric bias in metabolic measures; only hippocampal OEF exhibited a modest leftward asymmetry (P = 0.020, dz=0.42), whereas all other metabolic indices were symmetric (Supplementary Figure 3). Correspondingly, intersubject volumetric comparisons revealed significant rightward asymmetry in the amygdala (P < 0.001, dz=−0.65), a modest leftward asymmetry in the thalamus (P = 0.033, dz=0.38), and no significant asymmetry in hippocampal volumes (Supplementary Figure 4).

## 4. Discussion

The present study explored the effects of subjects’ age on OEF, CBF, and CMRO_2_ by means of the constrained quantitative BOLD MRI technique. Also examined were associations between age-related changes in these metabolic parameters and brain volume. Lastly, interhemispheric laterality was investigated for measures of oxygen metabolism and volume.

The results indicate that healthy older adults exhibit reduced CBF, along with greater OEF compared to their younger counterparts, both globally and regionally (e.g. in the medial temporal lobe (MTL), hippocampus, amygdala, thalamus, and frontal and parietal lobes). Reduced cerebral blood flow is a well-established feature of normal aging (Mokhber et al., 2021), as has been demonstrated previously in several PET and MRI studies (Catchlove et al., 2018; Aanerud et al., 2012; De Vis et al., 2015; Pantano et al., 1984; Goyal et al., 2017; Leenders et al., 1990; Lu et al., 2011; Yamaguchi et al., 1986). The greater OEF, according to Fick’s Principle, is a compensatory mechanism to maintain CMRO_2_ at levels comparable to the younger subjects, despite age-related reductions in blood flow. One early PET study demonstrated lower CBF and CMRO_2_ with little change in OEF (Leenders et al., 1990), however, this study was performed at low spatial resolution (16 mm), and may have been affected by partial volume effects. Nevertheless, decreases in CBF observed in the present study, particularly across gray matter, frontal, temporal, and parietal lobes are consistent with the findings from this early PET report.

Pantano et al., using ^15^O PET, found that CBF decreased linearly with age, while CMRO_2_ did not, which aligns with the present study (Pantano et al., 1984). However, when comparing the younger and older group directly, the authors found that gray matter CBF and CMRO_2_ decreased, while white matter oxygen metabolism remained relatively unaltered by age. This observation parallels the findings from the present study, which saw no difference in CBF or CMRO_2_ in white matter. In gray matter, CBF decreased significantly, whereas CMRO_2_ did not. More recently, Aanerud et al., using ^15^O-PET, showed that CBF declined with age, accompanied by a parallel increase in OEF in specific cortical and subcortical regions (Aanerud et al., 2012). Additionally, a meta-analysis of ^15^O PET papers reporting cerebral metabolic rate of glucose and oxygen consumption across the adult lifespan found that cerebral blood flow decreased with age (Goyal et al., 2017). It also showed CMRO_2_ trending negative, although the change was very minimal, and not nearly as drastic as the change in CMR_gluc_ (Goyal et al., 2017). Again, the present study demonstrates a similar trend in most brain regions, with the older group showing minimal difference in CMRO_2_ between age groups. Goyal et al. also compared the regional pattern in CMRO_2_ between younger and older subjects, and found that throughout the healthy adult lifespan, this pattern remained largely the same (Goyal et al., 2017). The present study confirms these findings using MRI, demonstrating similar regional differences in CMRO_2_ between age groups.

In the present study, CMRO_2_ remained largely unaffected by age across both age groups, consistent with prior PET and MRI findings showing that, while cerebral blood flow declines with age, oxygen metabolism tends to be relatively preserved or only modestly reduced (Catchlove et al., 2018; Aanerud et al., 2012). This stability underscores the remarkable capacity of the aging brain to maintain metabolic homeostasis through tightly coupled regulation of oxygen delivery and extraction. Mechanistically, this relative invariance of CMRO_2_ has been attributed to preservation of the tissue O_2_/CO_2_ ratio, which sustains mitochondrial phosphorylation potential and ATP synthesis even under reduced perfusion conditions (Buxton, 2024). Neurovascular coupling mechanisms dynamically adjust to maintain pO_2_, pCO_2_, and pH equilibrium across the microvascular network, thereby optimizing oxygen diffusion and capillary recruitment (DiNuzzo et al., 2024; Germuska et al., 2019). Experimental work has further shown that cerebrovascular autoregulation can preserve CMRO_2_ through selective modulation of CBF and OEF during altered oxygen availability (Carr et al., 2023). These findings suggest that the apparent stability of CMRO_2_ during aging reflects a finely balanced interplay between vascular adaptation, efficient oxygen diffusion, and neuronal metabolic flexibility.

One whole-brain MRI oximetry study using TRUST and phase contrast (PC) techniques demonstrated a reduction in CBF with an increase in CMRO_2_ in healthy aging (Lu et al., 2011). However, that study did not account for inter-individual differences in hematocrit and does not provide regional information to determine if specific areas of the brain differed in their metabolic response to aging. Jiang et al. developed an arterial-suppressed accelerated T_2_-relaxation-under-phase-contrast (AS-aTRUPC) MRI technique to estimate OEF in the basal veins of Rosenthal and the vein of Galen, which drain the MTL. They also obtained global OEF measurements in the superior sagittal sinus using conventional TRUST MRI for comparison. Their results support the findings of the present study, showing a significant increase in OEF, both globally and within the MTL, with increasing age (Jiang et al., 2023). Furthermore, that study also showed excellent agreement when comparing linear regression coefficients, with the present study finding a 0.143 % change per year, and Jiang et al. showing 0.100 %/year in the basal veins, and 0.146 %/year in the vein of Galen (Jiang et al., 2023). Whole-brain results also showed strong concordance (0.150 %/year versus 0.148 %/year) with the data in the present work.

In addition to the global effects, region-specific analyses within the MTL revealed notable differences in OEF relative to whole-brain values. In the younger group, MTL OEF was on average 2.3 % lower than whole brain (32.2 % versus 34.5 %). This value for MTL OEF is in close agreement with a prior ^15^O-PET study that investigated regional differences in cerebral oxygen metabolism, where parahippocampal gyrus OEF was found to average 33 % compared with values of 36–45 % in most cortical and subcortical regions (Ito et al., 2023). This regional disparity was even more pronounced for the hippocampus and amygdala, which both saw a more than a 5 % difference on average (34.5 % versus 29.1 % and 27.9 %, respectively). These results are again consistent with Jiang et al., who found the average OEF measured at the basal veins (23.9 %) to be significantly lower than the whole-brain value (33.3 %) (Jiang et al., 2023). While average whole-brain values were highly comparable between studies, Jiang et al. showed a larger difference between MTL and SSS OEF. However, because the basal veins drain the medial temporal lobe, including the hippocampus and amygdala, as well as adjacent structures, the extent to which it specifically represents MTL oxygenation remains uncertain. Furthermore, their use of assumed hematocrit values introduces additional uncertainty in OEF estimation, which may contribute to the small discrepancy observed between studies. Although CMRO_2_ remained essentially independent of age, a positive association between CMRO_2_ and age was observed in the amygdala within the MTL. This finding should be interpreted cautiously given the relatively small size of the amygdala and the potential influence of partial volume effects or segmentation variability, and the apparent sensitivity of this regional estimate to covariates such as sex. Nevertheless, the accompanying pattern of increased OEF with relatively preserved perfusion suggests that regional metabolic regulation within limbic structures may differ from that of larger cortical regions, warranting further investigation in larger cohorts.

In the older cohort, the difference between MTL and whole brain OEF was still evident (37.4 % vs 40.2 %). This was also true for the hippocampus and amygdala (37.2 and 38.3 versus 40.2 %, respectively). The values found in the latter structures are significantly higher than those reported by Yang et al., who used the QSM + qBOLD method to measure cerebral oxygen metabolism in Alzheimer’s Disease patients and age-matched controls (Yang et al., 2023). They reported mean OEF values in older adults of 27.9 % in the hippocampus and 27.2 % in the amygdala. There is likely a bias between the present method and the QQ method when measuring regional cerebral oxygen metabolism and comparing absolute results between the two methods is not straightforward. This bias may be attributed to the difference in R_2_’ measurement, the addition of CBVv as a prior constraint in constrained qBOLD, or the clustering algorithms used in the QQ method (Lee and Wehrli, 2022; Cho et al., 2021). The value for MTL OEF in the older cohort obtained in the present study agrees well with a prior ^15^O-PET study of Alzheimer’s Disease patients and healthy matched controls (Ishii et al., 1996) (37.4 % versus 35.2 %). Overall, the findings from the present study suggest that the MTL structures, particularly the hippocampus and amygdala, exhibit lower OEF than whole-brain averages, reflecting their distinct neurovascular architecture and metabolic efficiency, which may render them more vulnerable to hypoperfusion during aging.

Beyond MTL, age-related changes in oxygen metabolism were also evident in the frontal and parietal regions. These areas exhibited reduced CBF accompanied by modest increases in OEF, resulting in moderate negative associations of CMRO_2_ with age. The frontal and parietal cortices, which support higher-order cognitive integration, are particularly susceptible to age-related reductions in perfusion and vascular reactivity (De Vis et al., 2015; Tarumi and Zhang, 2018). This pattern of age-related metabolic changes may reflect the combined influence of vascular and neural factors. Systemic processes such as microvascular rarefaction and endothelial dysfunction contribute to global reductions in cerebral perfusion, while regionally reduced neuronal activity and synaptic density in the frontal and parietal cortices may further diminish cerebral blood flow and metabolism (Tarumi and Zhang, 2018).

In addition to the frontal and parietal cortices, age-related metabolic patterns were also observed in the occipital lobe. The occipital cortex suggested increased OEF with age, rising from 35.1 % in younger to 40.7 % in older adults. Prior PET studies have shown OEF to be significantly elevated in the occipital cortex compared to whole-brain values under resting conditions (49 % vs. 44 %) (Henriksen et al., 2021). However, the difference between occipital lobe OEF and whole brain in the present study was small (35.1 vs. 34.5 % in younger subjects, 40.7 vs. 40.2 in older subjects). One possible reason for this discrepancy is visual stimulus activation. While most subjects analyzed in Henriksen et al. were asked to keep their eyes open during scanning (Henriksen et al., 2021), the present study’s protocol was not standardized. In another PET study of regional OEF differences where subjects were awake but had their eyes closed, there were, on average, deviations in OEF of ~1.1 in the visual cortex (Raichle et al., 2001). Furthermore, average-whole brain OEF was 40 % with a mean age of ~40 years, which agrees well with our findings. The occipital lobe OEF in a group of healthy older adults (mean age ~60 years) found in Ishii et al. also agreed well with the present results (41.3 vs 40.7 %) (Ishii et al., 1996). Importantly, Ishii et al. did not find significant differences in OEF in the occipital lobe compared to other areas of the human cortex (Ishii et al., 1996).

Subcortical regions such as the thalamus and striatum also exhibited age-related declines in perfusion with compensatory increases in oxygen extraction, following a pattern similar to that seen in cortical regions. Prior PET studies have also demonstrated regional coupling between cerebral perfusion and oxygen metabolism across cortical and subcortical structures, including the basal ganglia, alongside significant regional variability in oxygen extraction (Henriksen et al., 2021). Structural MRI studies report progressive thalamic atrophy and microstructural alterations across the adult lifespan (Hughes et al., 2012; Choi et al., 2022), while metabolic imaging reveals decreased N-acetyl-aspartate and creatine with increased myo-inositol, indicating concomitant neuronal and glial metabolic changes (Ahlswede et al., 2021). These converging findings suggest that perfusion declines in this region reflect a combination of neural and cerebrovascular factors rather than purely vascular vulnerability. Subcortical nuclei such as the thalamus may preserve oxygen metabolism through adaptive adjustments in oxygen extraction and neurovascular coupling like those observed in cortical association areas.

For the three measures of brain metabolism (OEF, CBF, CMRO_2_), laterality indices for the hippocampus and amygdala were consistently small across both age groups, indicating minimal hemispheric asymmetry. This finding is in line with the results of Yang et al., who reported negligible left-right differences in OEF, CBF, and CMRO_2_ within these regions among healthy older control subjects (Yang et al., 2023). Similarly, Jiang et al. found no significant hemispheric bias in OEF when comparing measurements obtained from the right and left basal veins (Jiang et al., 2023). While most of the present results align with these findings, a modest leftward asymmetry was detected in the amygdala when data were separated by age group, and a subtle leftward bias of the hippocampal OEF when analyzed across the whole group, however, these effects were very small (<0.1 %). In terms of structure, volumetric analyses revealed modest rightward asymmetry of the amygdala and slight leftward asymmetry of the thalamus in both younger and older adults, whereas hippocampal volumes were largely symmetric. These patterns are broadly consistent with prior structural MRI research reporting modest rightward volumetric asymmetry of the hippocampus and amygdala (Pedraza et al., 2004) and a small leftward asymmetry of the thalamus in healthy adults (Lubben et al., 2021). Large-scale population studies have further demonstrated that such hemispheric asymmetries are common features of normal brain organization, with magnitudes falling within the normal anatomical variability observed in healthy cohorts (Kong et al., 2018).

In agreement with previous studies, global and regional brain volumes were smaller in the older compared to the younger subjects, reflecting characteristic atrophic changes associated with normal aging. Numerous MRI studies have consistently demonstrated widespread reductions in both cortical and subcortical gray matter volumes with advancing age (Long et al., 2012; Sele et al., 2020; Zheng et al., 2019). The most pronounced declines typically occur in the frontal, parietal, and medial temporal regions, including the hippocampus and amygdala, which are known to exhibit accelerated volumetric loss across mid- to late adulthood (Long et al., 2012; Sele et al., 2020). Subcortical structures such as the thalamus, caudate, and putamen also undergo progressive shrinkage, though at slightly slower rates (Zheng et al., 2019; Király et al., 2016). The present results therefore align with the broader neuroimaging literature indicating that brain aging is characterized by diffuse, yet regionally variable, volume loss, most notably in association cortices and limbic regions.

The present findings of preserved CMRO_2_ yet elevated OEF in the context of reduced CBF align with emerging evidence that cerebral oxygen metabolism metrics are not only markers of physiological aging but may have clinical relevance in assessing brain health. In cohorts of older adults, MRI-derived OEF and CMRO_2_ have been linked to cognitive performance, with lower OEF and CMRO_2_ associated with worse executive function and neuropsychological outcomes, particularly among individuals with genetic risk factors or existing cognitive impairment, underscoring their potential utility in early detection of neurovascular vulnerability (Robb et al., 2022). Moreover, previous work demonstrated that age-related increases in OEF reflect alterations in the coupling between cerebral oxygen supply and metabolic demand (Lu et al., 2011), a physiological framework that has been further extended by recent quantitative MRI studies to show that differences in cerebral oxygen extraction can help distinguish pathological conditions such as Alzheimer’s disease and vascular cognitive impairment (Jiang et al., 2020). These findings support the concept that noninvasive MRI measures of cerebral oxygen metabolism could serve as biomarkers of neurovascular and metabolic integrity in aging, with applications in identifying individuals at elevated risk for cognitive decline, evaluating the efficacy of vascular or metabolic interventions, and guiding clinical decision-making in age-related neurological disorders.

While the combined constrained qBOLD–ASL technique offers a powerful noninvasive framework for simultaneous mapping of OEF, CBF, and CMRO_2_, several methodological considerations should be noted. First, constrained qBOLD depends on accurate estimation of magnetic susceptibility and venous cerebral blood volume from AUSFIDE and VS-VSL sequences, both of which are sensitive to macroscopic field inhomogeneity and residual motion artifacts, potentially affecting parameter precision. Although the constraint-based model mitigates noise amplification and improves quantitative stability compared with conventional qBOLD or QSM+qBOLD approaches, partial volume effects and limited spatial resolution may still introduce variability in small structures such as the amygdala and hippocampus. Partial volume effects are of particular concern in the older cohort, where brain atrophy is evident. Meltzer et al. demonstrated the effect of brain atrophy on PET CBF measurements, where the inverse correlation between CBF and age was only significant before partial volume correction (Meltzer et al., 2000). Furthermore, studies have shown that PET OEF measurements can be affected by the volume fraction of tissue type in a voxel (Fan et al., 2020). However, this is driven by the dependency on the CBF measurement for accurate quantification (Fan et al., 2020). Constrained qBOLD’s measurement of OEF does not depend on CBF, and the smaller in-plane voxel size (1.5 mm) helps mitigate potential partial volume effects. However, partial volume effects in CBF may still be evident. Another limitation to CBF measurement with ASL is the selection of the post-labeling delay (PLD) time. While a slightly longer PLD of 2 s was chosen for this study, which was recommended for older patients (Dai et al., 2017), selecting a PLD time that is too short may underestimate CBF in some participants.

Previous MRI studies have shown moderate test–retest repeatability of oxygen extraction and metabolic rate measurements at the level of global or gray-matter averages (Lajoie et al., 2016), but explicit evaluation of reproducibility in small subcortical regions has not yet been established. This underscores the need for future work to directly assess measurement stability in small regions and to refine acquisition and processing strategies to enhance regional quantification. Second, the use of fixed assumptions for arterial oxygen saturation (SaO_2_) may introduce intersubject variability in CMRO_2_ estimates, though SaO_2_ is generally well-maintained in healthy individuals under resting conditions. In addition, end-tidal CO_2_ (EtCO_2_) was not measured, which has been shown to affect with OEF (Jiang et al., 2020). Lastly, this study’s cross-sectional design precludes direct inference of longitudinal metabolic trajectories across the adult lifespan. Future work employing larger, longitudinal cohorts and incorporating individualized calibration of arterial saturation and EtCO_2_ will further strengthen the translational applicability of constrained qBOLD-derived metrics.

In conclusion, this study demonstrates that age-related reductions in cerebral blood flow are accompanied by compensatory increases in oxygen extraction, resulting in largely preserved oxygen metabolism across the adult lifespan. The spatially resolved constrained qBOLD approach proved effective in detecting regional variations in oxygen metabolism, particularly within the medial temporal, frontal, parietal, and thalamic regions, supporting its value as a quantitative imaging biomarker for cerebrovascular aging. These findings lay the groundwork for future applications of constrained qBOLD in characterizing neurovascular and metabolic alterations in age-related and neurodegenerative disorders.

## Supplementary Material

1

Supplementary material associated with this article can be found, in the online version, at doi:10.1016/j.neuroimage.2026.121967.

## Figures and Tables

**Table 1:** Summary of participant demographics.

Age Group	Younger adults (21–35)	Older adults (50–90)	P- value
Number of Participants	15	19	N/A
Age (years)	27.7 ± 3.4	68.3 ± 10.9	**<0.001**
Weight (kg)	78.5 ± 16.6	71.4 ± 15.7	0.21
Body Mass Index (kg/m^2^)	25.2 ± 5.5	24.3 ± 4.4	0.61
Hematocrit (%)	43.8 ± 3.5	43.4 ± 5.3	0.84

**Table 2:** Whole-brain and regional metabolic parameters across age groups. Values represent mean ± standard deviation for each region in younger and older adults. P-values are from independent two-sample *t*-tests (^†^) when normality assumptions were satisfied and Mann-Whitney U (^§^) otherwise.

Age Group	OEF (%)			CBF (mL/100 g/min)			CMRO_2_ (μmolO_2_/100 g/min)		
	21–35 (N = 15)	50–90 (N = 19)	P-value	21–35 (N = 15)	50–90 (N = 19)	P-value	21–35 (N = 15)	50–90 (N = 19)	P-value
**WB**	34.5 ± 4.6	40.2 ± 5.1	**0.002** ^†^	48.3 ± 8.3	39.5 ± 10.3	**0.011** ^†^	150.7 ± 30.0	144.0 ± 31.8	0.537^†^
**GM**	34.9 ± 4.2	40.3 ± 4.7	**0.002** ^†^	57.1 ± 10.4	47.7 ± 12.9	**0.028** ^†^	184.8 ± 36.3	171.6 ± 38.4	0.317^†^
**WM**	33.5 ± 5.0	39.5 ± 5.6	**0.003** ^†^	30.3 ± 7.1	28.2 ± 8.0	0.425^†^	91.5 ± 23.9	101.1 ± 30.6	0.325^†^
**MTL**	32.2 ± 5.1	37.4 ± 5.9	**0.011** ^†^	45.5 ± 9.2	37.0 ± 10.6	**0.001** ^§^	129.8 ± 31.1	121.3 ± 22.7	0.365^†^
**HC**	29.1 ± 6.1	37.2 ± 7.0	**0.001** ^†^	45.1 ± 11.5	37.0 ± 10.9	**0.011** ^§^	116.4 ± 36.0	121.5 ± 26.3	0.319^§^
**AG**	27.9 ± 6.1	38.3 ± 8.6	**<0.001** ^†^	39.4 ± 8.9	36.9 ± 12.0	0.202^§^	97.5 ± 31.6	126.6 ± 35.9	**0.011** ^§^
**FL**	34.7 ± 5.0	38.7 ± 5.2	**0.028** ^†^	57.6 ± 11.3	44.3 ± 11.8	**0.002** ^†^	177.1 ± 39.4	153.7 ± 40.1	0.098^†^
**PL**	38.2 ± 5.5	43.9 ± 5.1	**0.004** ^†^	62.1 ± 11.3	46.3 ± 13.7	**0.001** ^†^	211.6 ± 46.8	180.5 ± 44.6	0.057^†^
**THL**	32.5 ± 7.1	40.5 ± 6.4	**0.002** ^†^	38.9 ± 8.1	31.8 ± 8.4	**0.018** ^†^	112.8 ± 29.2	116.5 ± 32.5	0.734^†^
**OCC**	35.1 ± 5.3	40.7 ± 6.3	**0.009** ^†^	59.1 ± 11.1	45.1 ± 14.0	**0.004** ^†^	185.6 ± 41.5	161.3 ± 36.3	0.079^†^
**STR**	36.2 ± 5.5	42.7 ± 5.3	**0.001** ^†^	36.0 ± 10.5	28.9 ± 7.5	**0.019** ^§^	116.1 ± 33.4	111.7 ± 26.6	0.945^§^

**OEF**; oxygen extraction fraction, **CBF**; cerebral blood flow, **CMRO_2_**; cerebral metabolic rate of oxygen, **WB**; whole brain, **GM**; gray matter, **WM**; white matter, **MTL**; medial temporal lobe, **HC**; hippocampus, **AG**; amygdala, **FL**; frontal lobe, **PL**; parietal lobe, **THL**; thalamus, **OCC**; occipital, **STR**; striatum.

**Table 3:** OEF, CBF and CMRO_2_ versus age across brain regions. Values represent regression coefficients (B), corresponding standard errors (SE), and two-tailed p-values from multivariate linear regression models examining the association between OEF, CBF and CMRO_2_ and age. Coefficients and standard errors are expressed as percentage change per year of age. Positive slopes indicate increasing values with advancing age.

Region	OEF			CBF			CMRO2		
	Coefficient (%/year)	SE (%/year)	P-value	Coefficient (%/year)	SE (%/year)	P-value	Coefficient (%/year)	SE (%/year)	P-value
**WB**	0.150	0.037	**<0.001**	−0.207	0.074	**0.009**	−0.157	0.239	0.517
**GM**	0.128	0.036	**0.001**	−0.250	0.091	**0.01**	−0.450	0.282	0.121
**WM**	0.164	0.040	**<0.001**	−0.051	0.059	0.389	0.228	0.223	0.315
**MTL**	0.141	0.041	**0.002**	−0.208	0.079	**0.014**	−0.206	0.210	0.333
**HC**	0.222	0.047	**<0.001**	−0.187	0.090	**0.047**	0.169	0.237	0.481
**AG**	0.285	0.052	**<0.001**	−0.026	0.086	0.764	0.830	0.238	**0.002**
**FL**	0.089	0.041	**0.04**	−0.327	0.088	**<0.001**	−0.682	0.298	**0.029**
**PL**	0.149	0.041	**<0.001**	−0.385	0.099	**<0.001**	−0.795	0.357	**0.034**
**THL**	0.211	0.050	**0.002**	−0.178	0.062	**0.007**	0.056	0.252	0.825
**OCC**	0.162	0.043	**<0.001**	−0.344	0.101	**0.002**	−0.565	0.308	0.076
**STR**	0.169	0.039	**<0.001**	−0.159	0.071	**0.032**	−0.085	0.239	0.726

**SE**; standard error, **WB**; whole brain, **GM**; gray matter, **WM**; white matter, **MTL**; medial temporal lobe, **HC**; hippocampus, **AG**; amygdala, **FL**; frontal lobe, **PL**; parietal lobe, **THL**; thalamus, **OCC**; occipital, **STR**; striatum.

**Table 4:** Regional differences in metabolic parameters relative to whole-brain averages across age groups. Values represent mean differences (Δ = region – whole brain) ± standard deviation with corresponding 95 % confidence intervals, computed using paired *t*-test output for consistency across regions. Positive values indicate higher regional vascular-metabolic measures than whole-brain means, while negative values indicate lower values. Statistical comparisons were performed using two-sided paired-sample *t*-tests (^†^) when normality assumptions were satisfied and Wilcoxon signed-rank test (^§^) otherwise, with Z-statistics reported for non-parametric results.

Region	OEF (%)			CBF (mL/100 g/min)			CMRO_2_ (μmolO_2_/100 g/min)		
	Δ	95 % CI	P-value	Δ	95 % CI	P-value	Δ	95 % CI	P-value
**Age group: 21–35 (N = 15)**									
**GM**	0.5 ± 1.2	−0.2, 1.1	0.150^†^	8.7 ± 6.1	5.4, 12.1	**<0.001** ^†^	34.1 ± 26.3	19.5, 48.6	**<0.001** ^†^
**WM**	−1.0 ± 1.1	−1.6, −0.4	**0.004** ^†^	−18.0 ± 6.1	−21.4, −14.6	**<0.001** ^†^	−59.3 ± 20.5	−70.6, −47.9	**<0.001** ^†^
**MTL**	−2.3 ± 2.7	−3.8, −0.7	**0.006** ^†^	−2.8 ± 5.5	−5.9, 0.2	0.100^§^ (Z=−1.65)	−20.9 ± 19.1	−31.5, −10.3	**0.001** ^†^
**HC**	−5.4 ± 2.9	−7.0, −3.8	**<0.001** ^†^	−3.2 ± 6.9	−7.0, 0.6	0.100^§^ (Z=−1.65)	−34.4 ± 27.2	−49.4, −19.3	**0.002**^§^ (Z=−3.07)
**AG**	−6.6 ± 5.6	−9.7, −3.4	**<0.001** ^†^	−8.9 ± 4.3	−11.3, −6.5	**0.001**^§^ (Z=−3.41)	−53.2 ± 31.8	−70.8, −35.6	**0.001**^§^ (Z=−3.41)
**FL**	0.2 ± 1.4	−0.6, 1.0	0.561^†^	9.3 ± 4.3	6.9, 11.7	**0.001**^§^ (Z=−3.41)	26.4 ± 14.2	18.5, 34.3	**<0.001** ^†^
**PL**	3.7 ± 2.3	2.5, 5.0	**<0.001** ^†^	13.7 ± 5.0	10.9, 16.5	**0.001**^§^ (Z=−3.41)	60.9 ± 21.3	49.1, 72.7	**<0.001** ^†^
**THL**	−2.0 ± 3.4	−3.9, −0.1	**0.038** ^†^	−9.4 ± 6.2	−12.8, −5.9	**<0.001** ^†^	−37.9 ± 18.9	−48.3, −27.4	**<0.001** ^†^
**OCC**	0.6 ± 2.2	−0.6, 1.8	0.303^†^	10.7 ± 5.0	8.0, 13.5	**0.001**^§^ (Z=−3.41)	34.8 ± 18.5	24.6, 45.1	**<0.001** ^†^
**STR**	1.7 ± 2.4	0.4, 3.0	**0.014** ^†^	−12.3 ± 5.7	−15.5, −9.2	**0.001**^§^ (Z=−3.41)	−34.7 ± 21.6	−46.6, −22.7	**0.001**^§^ (Z=−3.41)
**Age group: 50–90 (N = 19)**									
**GM**	0.1 ± 1.8	−0.8, 0.9	0.901^†^	8.2 ± 11.5	2.7, 13.7	**0.002**^§^ (Z=−3.10)	27.6 ± 35.9	10.2, 44.9	**0.004** ^†^
**WM**	−0.7 ± 1.6	−1.5, 0.1	0.085^†^	−11.3 ± 5.7	−14.0, −8.5	**0.001** ^†^	−42.9 ± 17.6	−51.4, −34.5	**<0.001** ^†^
**MTL**	−2.8 ± 3.0	−4.3, −1.3	**0.001** ^†^	−2.5 ± 2.9	−3.9, −1.1	**0.005**^§^ (Z=−2.78)	−22.7 ± 14.3	−29.6, −15.8	**<0.001** ^†^
**HC**	−3.0 ± 3.9	−4.9, −1.1	**0.004** ^†^	−2.5 ± 5.2	−5.0, 0.0	0.077^§^ (Z=−1.77)	−22.5 ± 17.6	−31.0, −14.0	**<0.001** ^†^
**AG**	−1.9 ± 6.7	−5.1, 1.4	0.245^†^	−2.6 ± 10.1	−7.5, 2.3	**0.008**^§^ (Z=−2.66)	−17.4 ± 33.5	−33.6, −1.3	**0.036^†^**
**FL**	−1.5 ± 2.8	−2.8, −0.1	**0.036** ^†^	4.8 ± 3.1	3.3, 6.3	**<0.001** ^†^	9.6 ± 17.8	1.0, 18.3	**0.030** ^†^
**PL**	3.7 ± 2.4	2.6, 4.9	**<0.001** ^†^	6.8 ± 5.5	4.2, 9.5	**<0.001**^§^ (Z=−3.50)	36.5 ± 24.1	24.8, 48.1	**<0.001** ^†^
**THL**	0.3 ± 2.8	−1.1, 1.6	0.692^†^	−7.6 ± 6.2	−10.6, −4.6	**<0.001** ^†^	−27.5 ± 18.9	−36.6, −18.4	**<0.001** ^†^
**OCC**	0.5 ± 2.9	−0.9, 1.9	0.465^†^	5.7 ± 5.6	3.0, 8.4	**0.001**^§^ (Z=−3.26)	17.3 ± 19.9	7.7, 26.9	**0.001** ^†^
**STR**	2.5 ± 2.7	1.2, 3.8	**0.001** ^†^	−10.5 ± 3.8	−12.3, −8.7	**<0.001** ^†^	−32.3 ± 12.9	−38.6, −26.1	**<0.001** ^†^

**OEF**; oxygen extraction fraction, **CBF**; cerebral blood flow, **CMRO_2_**; cerebral metabolic rate of oxygen, **MTL**; medial temporal lobe, **GM**; gray matter, **WM**; white matter, **HC**; hippocampus, **AG**; amygdala, **FL**; frontal lobe, **PL**; parietal lobe, **THL**; thalamus, **OCC**; occipital, **STR**; striatum.

**Table 5:** Whole-brain and regional volumetric parameters across age groups. Values represent mean volumes (mL) ± standard deviation for each brain region in younger and older adults. P-values are from independent two-sample *t*-tests (^†^) when normality assumptions were satisfied and Mann-Whitney U (^§^) otherwise.

Volumes (mL)	Age group: 21–35 (N = 15)	Age group: 50–90 (N = 19)	P- value
**WB**	1216.4 ± 97.2	1075.0 ± 125.4	**0.001** ^†^
**GM**	685.2 ± 72.1	586.6 ± 86.3	**0.001** ^†^
**GM/WB**	0.56 ± 0.04	0.54 ± 0.05	**0.025** ^§^
**WM**	493.9 ± 48.8	457.9 ± 62.4	0.076^†^
**WM/WB**	0.41 ± 0.02	0.43 ± 0.02	**0.012** ^†^
**HC**	8.6 ± 0.9	7.1 ± 1.5	**0.002** ^§^
**AG**	3.0 ± 0.6	2.7 ± 0.8	0.104^§^
**THL**	19.9 ± 2.1	17.6 ± 3.3	**0.029** ^†^

**WB**; whole brain, **GM**; gray matter, **WM**; white matter, **HC**; hippocampus, **AG**; amygdala, **THL**; thalamus.

**Table 6:** Laterality indices of metabolic parameters in the hippocampus and amygdala across age groups. Values represent mean laterality indices (LI ± standard deviation) with corresponding 95 % confidence intervals computed using *t*-test output for consistency. P-values are from one-sample *t*-tests (^†^) when normality assumptions were satisfied and Wilcoxon signed-rank tests (^§^) otherwise, with Z statistics reported for non-parametric results. Positive LI values indicate rightward asymmetry, while negative LI values indicate leftward asymmetry. LI was calculated as (Right − Left)/(Right + Left).

Region	OEF (%)			CBF (mL/100 g/min)			CMRO_2_ (μmolO_2_/100 g/min)		
	Mean ± SD	95 % CI	P-value	Mean ± SD	95 % CI	P-value	Mean ± SD	95 % CI	P-value
**Age group: 21–35 (N = 15)**									
**HC**	−0.02 ± 0.05	−0.05, 0.00	0.053^†^	0.01 ± 0.06	−0.02, 0.05	0.609^§^ (Z=−0.51)	−0.01 ± 0.09	−0.06, 0.04	0.100^§^ (Z=−1.65)
**AG**	0.04 ± 0.13	−0.03, 0.11	0.256^†^	0.03 ± 0.08	−0.01, 0.07	0.281^§^ (Z=−1.08)	0.07 ± 0.16	−0.02, 0.16	0.105^†^
**Age group: 50–90 (N = 19)**									
**HC**	−0.01 ± 0.04	−0.03, 0.01	0.309^†^	−0.02 ± 0.05	−0.04, 0.01	0.153^†^	−0.03 ± 0.06	−0.06, 0.00	0.086^†^
**AG**	−0.03 ± 0.05	−0.05, 0.00	**0.006**^§^ (Z=−2.74)	0.00 ± 0.09	−0.04, 0.04	0.982^†^	−0.03 ± 0.11	−0.08, 0.02	0.265^†^

**OEF**; oxygen extraction fraction, **CBF**; cerebral blood flow, **CMRO_2_**; cerebral metabolism rate of oxygen, **LI**; laterality index, **HC**; hippocampus, **AG**; amygdala.

## Data Availability

All data supporting the findings of this study are available within the paper and its supplementary material.
