# Supplementary material for: Age dependence of brain oxygen metabolism in adults assessed by 3D constrained quantitative BOLD MRI
Source: Neuroimage. Author manuscript; Available in PMC 2026 Jun 8. (PMC13245583; doi:10.1016/j.neuroimage.2026.121967)

**Supplementary Material**

**Supplementary Table 1.** **Linear regression results for regional brain volume normalized to intracranial volume versus age across brain regions.** Values represent regression coefficients (B) and corresponding standard errors, 95% confidence intervals, and two-tailed p-values from models examining the association between normalized regional brain volume (%ICV) and age. Coefficients and standard errors are expressed as percent change per year of age. Negative slopes indicate decreasing volume percentage with advancing age.

| **Region** | **Coefficient (%ICV/year)** | **Standard error (%ICV/year)** | **P-value** |
| --- | --- | --- | --- |
| **WB** | -0.002 | <0.001 | **<0.001** |
| **GM** | -0.044 | 0.025 | 0.090 |
| **WM** | 0.042 | 0.025 | 0.108 |
| **HC** | -0.002 | 0.001 | **0.009** |
| **AG** | -0.001 | <0.001 | 0.105 |
| **THL** | 0.003 | 0.003 | 0.377 |

**ICV**; intracranial volume**, WB**; whole brain, **GM**; gray matter, **WM**; white matter, **HC**; hippocampus, **AG**; amygdala, **THL**; thalamus.

| **Region** | **Right (mL)** | **Left (mL)** | **LI** | **95% CI** | **P-value** |
| --- | --- | --- | --- | --- | --- |
| **Age group: 21-35 (N=15)** | | | | | |
| **HC** | 4.3 ± 0.5 | 4.4 ± 0.5 | -0.01 ± 0.03 | -0.03, 0.00 | 0.147^†^ |
| **AG** | 1.6 ± 0.3 | 1.4 ± 0.3 | 0.05 ± 0.08 | 0.01, 0.10 | **0.021**^†^ |
| **THL** | 9.7 ± 1.1 | 10.2 ± 1.1 | -0.02 ± 0.03 | -0.4, -0.01 | **0.011**^†^ |
| **Age group: 50-90 (N=19)** | | | | | |
| **HC** | 3.5 ± 0.8 | 3.5 ± 0.9 | 0.00 ± 0.15 | -0.07, 0.07 | 0.355^§^ (Z=-0.93) |
| **AG** | 1.4 ± 0.4 | 1.2 ± 0.4 | 0.10 ± 0.15 | 0.03, 0.17 | **0.004**^§^ (Z=-2.90) |
| **THL** | 8.4 ± 1.3 | 9.2 ± 2.5 | -0.03 ± 0.08 | -0.07, 0.00 | **0.016**^§^ (Z=-2.41) |

**Supplementary Table 2.** **Hemispheric volume comparison and laterality indices across age groups.** Values represent mean volumes (mL) for right and left hemispheric regions, with corresponding laterality indices (LI ± standard deviation) and 95% confidence intervals computed using t-test output for consistency. P-values are from one-sample t-tests (^†^) when normality assumptions were satisfied and Wilcoxon signed-rank tests (^§^) otherwise, with Z statistics reported for non-parametric results. Positive LI values indicate rightward asymmetry, while negative LI values indicate leftward asymmetry. LI was calculated as (Right−Left)/(Right+Left).

**LI**; laterality index, **HC**; hippocampus, **AG**; amygdala, **THL**; thalamus.

**Supplementary Figure 1. Laterality indices for neurometabolic parameters in the amygdala and hippocampus.** No significant hemispheric asymmetry was observed, except for a slight leftward bias in hippocampal OEF.


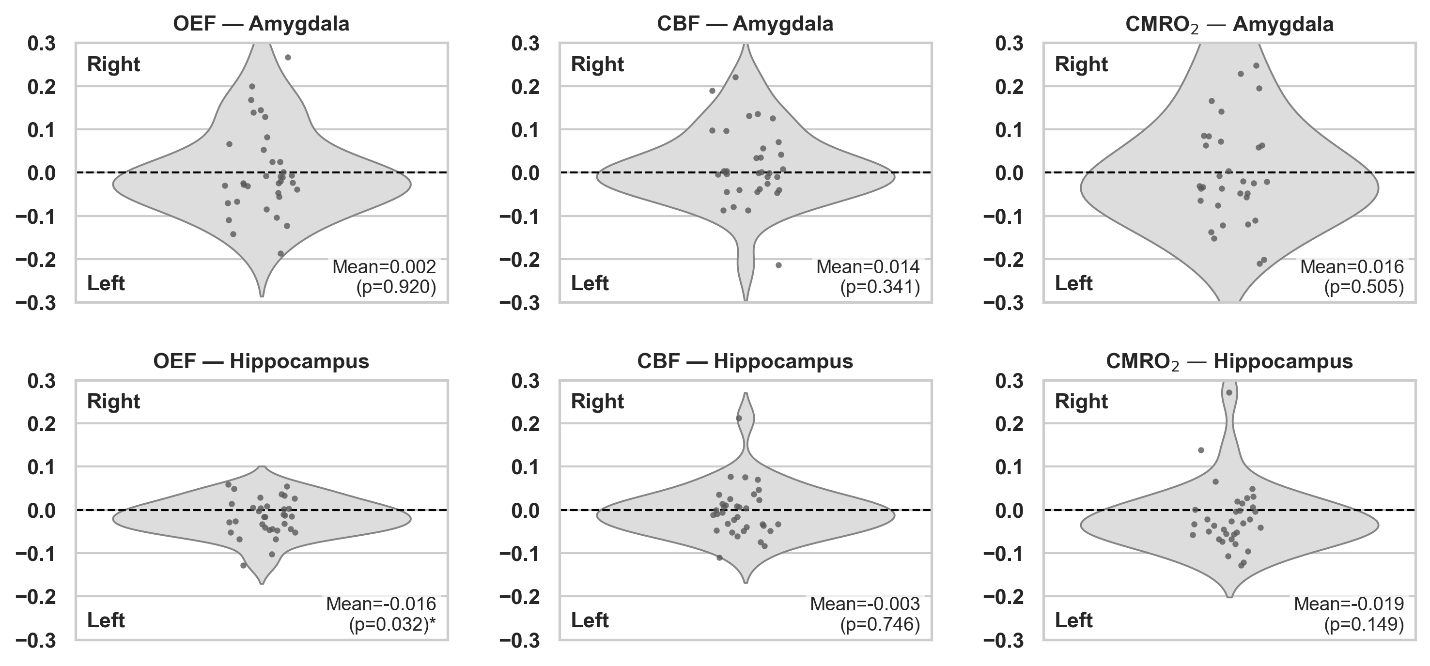


**Supplementary Figure 2. Laterality indices for regional brain volumes in the hippocampus, amygdala, and thalamus.** Significant rightward asymmetry was observed in the amygdala and leftward asymmetry in the thalamus, while no hemispheric difference was found in the hippocampus.


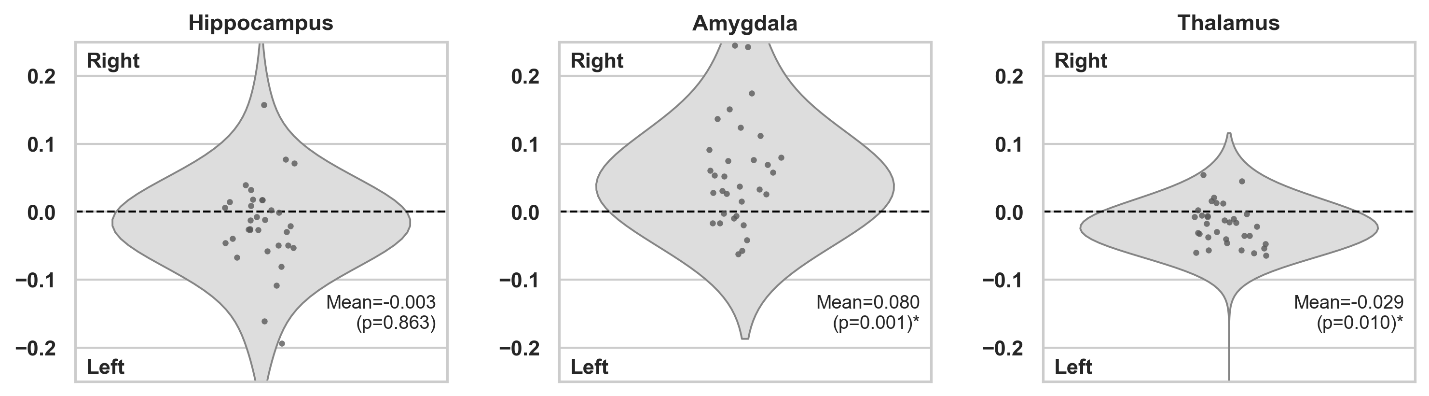


**Supplementary Figure 3. Within-subject laterality of regional metabolic parameters in the amygdala and hippocampus.** No significant hemispheric differences were observed in the amygdala, while the hippocampus showed higher OEF and CMRO_2_ in the right hemisphere.


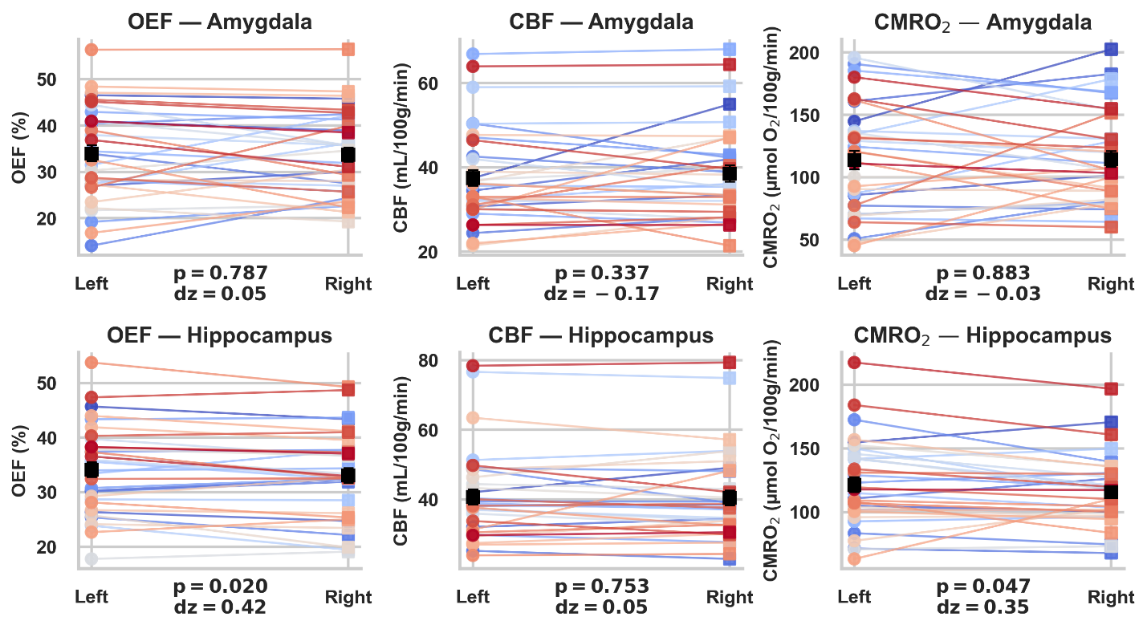


**Supplementary Figure 4. Within-subject laterality of regional brain volumes in the amygdala, hippocampus, and thalamus.** Significant rightward asymmetry was observed in the amygdala and leftward asymmetry in the thalamus, while hippocampal volumes showed no hemispheric difference.


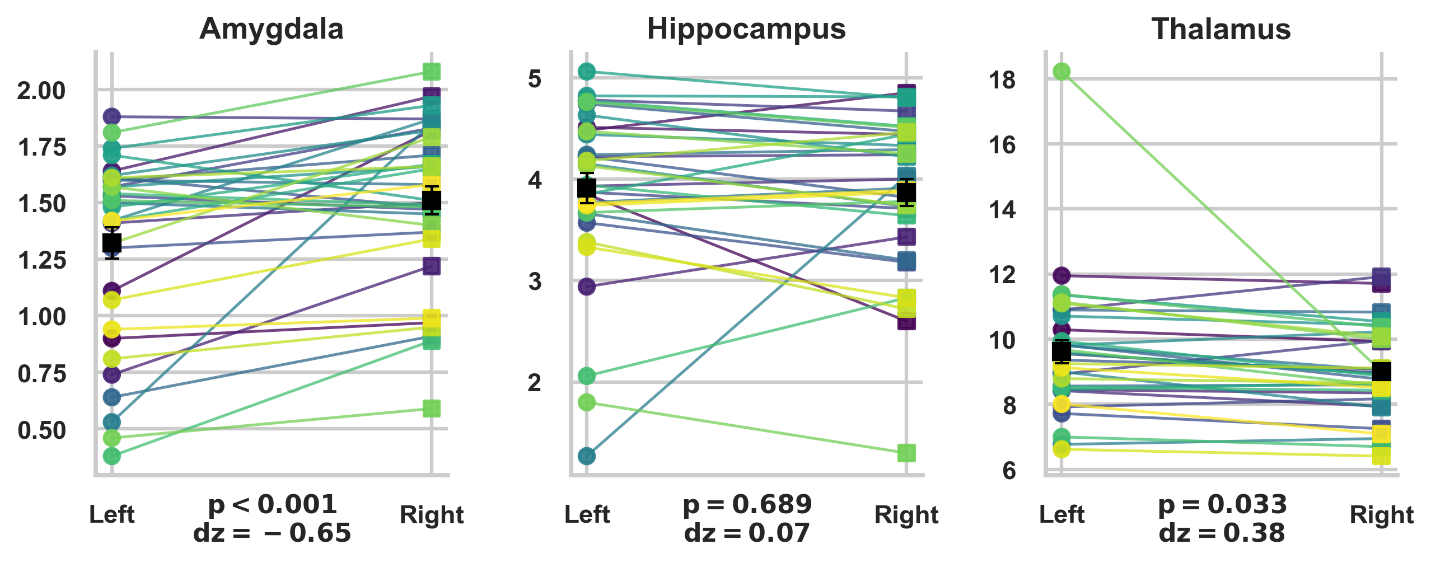

Supplement: 1 [file NIHMS2177639-supplement-1.docx]
